# Synthesis of AAB‐Stacked Single‐Crystal Graphene/hBN/Graphene Trilayer van der Waals Heterostructures by In Situ CVD

**DOI:** 10.1002/advs.202201324

**Published:** 2022-05-26

**Authors:** Bo Tian, Junzhu Li, Mingguang Chen, Haocong Dong, Xixiang Zhang

**Affiliations:** ^1^ Physical Science and Engineering Division King Abdullah University of Science and Technology (KAUST) Thuwal 23955–6900 Saudi Arabia; ^2^ Eleven‐Dimensional Nanomaterial Research Institute Xiamen 361000 P. R. China

**Keywords:** AAB‐stacking, graphene, hBN, in situ CVD, van der Waals heterostructures

## Abstract

van der Waals heterostructures based on graphene and hBN layers with different stacking modes are receiving considerable attention because of their potential application in fundamental physics. However, conventional exfoliation fabrication methods and layer‐by‐layer transfer techniques have various limitations. The CVD synthesis of high‐quality large‐area graphene and hBN multilayer heterostructures is essential for the advancement of new physics. Herein, the authors propose an in situ CVD growth strategy for synthesizing wafer‐scale AAB‐stacked single‐crystal graphene/hBN/graphene trilayer van der Waals heterostructures. Single‐crystal CuNi(111) alloys are prepared on sapphire, followed by the pre‐dissolution of carbon atoms. Single‐crystal monolayer hBN is synthesized on a plasma‐cleaned CuNi(111) surface. Then, a single‐crystal monolayer graphene is epitaxially grown onto the hBN surface to form graphene/hBN bilayer heterostructures. A controlled decrease in the growth temperature allows the carbon atoms to precipitate out of the CuNi(111) alloy to form single‐crystal graphene at the interface between hBN and CuNi(111), thereby producing graphene/hBN/graphene trilayer van der Waals heterostructures. The stacking modes between as‐grown 2D layers are investigated through Raman spectroscopy and transmission electron microscopy. This study provides an in situ CVD approach to directly synthesize large‐scale single‐crystal low‐dimensional van der Waals heterostructures and facilitates their application in future 2D‐material‐based integrated circuits.

## Graphical abstract

An in situ CVD synthesis of high‐quality single‐crystal graphene/hBN/graphene trilayer van der Waals heterostructures is presented here. A single‐crystal CuNi(111) film is produced on sapphire and then subjected to carbon dissolution. Single‐crystal monolayer hBN and graphene are then synthesized. Carbon atoms diffuse to the hBN–CuNi(111) interface and form graphene. This study paves the way for future 2D‐material‐based large‐area integrated circuits.

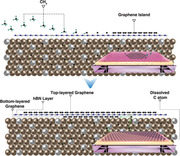

## 1 Introduction

2D van der Waals heterostructures are currently regarded as promising materials owing to their special electronic structures.^[^ ^1^, ^2^, ^3^, ^4^, ^5^ ^]^ The different stacking of graphene and hexagonal boron nitride (hBN) layers form heterostructures exhibiting novel physical properties.^[^ ^6^, ^7^, ^8^, ^9^, ^10^, ^11^, ^12^, ^13^, ^14^ ^]^ The van der Waals heterostructures consisting of graphene and hBN used in most studies were fabricated using mechanical exfoliation and layer‐by‐layer transfer techniques.^[^ ^10^, ^11^, ^12^, ^13^ ^]^ The exfoliation of single or multilayer graphene and hBN from bulk materials preserve the high crystal quality and intrinsic physical properties of thin materials. However, disadvantages such as small sample size and interlayer chemical contamination limit their applications in scientific research and industrial applications.^[^ ^15^, ^16^ ^]^ The chemical vapor deposition (CVD) synthetic technology has facilitated the production of high‐quality, large‐area, single‐crystal monolayer graphene and hBN.^[^ ^17^, ^18^, ^19^, ^20^, ^21^, ^22^, ^23^, ^24^ ^]^ Many researchers have synthesized graphene and hBN heterostructures using the CVD method.^[^ ^4^, ^25^, ^26^, ^27^ ^]^ Currently, graphene/hBN, hBN/graphene, and hBN/graphene/hBN have been grown on Cu substrates in situ via CVD.^[^ ^28^, ^29^, ^30^ ^]^ Although the graphene/hBN/graphene heterostructure is considered an ideal system to investigate thermoelectric transport in the cross‐plane direction and has demonstrated significant application in tunnel transistors,^[^ ^31^, ^32^ ^]^ the in situ CVD growth of high‐quality graphene/hBN/graphene heterostructures has been rarely reported. Hence, there is an urgent need for a CVD growth method for fabricating large‐area single‐crystal graphene/hBN/graphene heterostructures to be used in both fundamental research and industrial applications.

**Figure 1:**
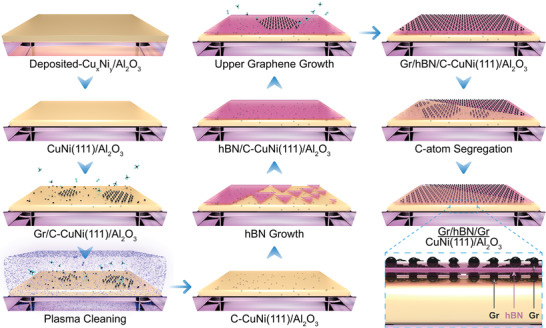
Schematic illustration of the in‐situ CVD fabrication process of the trilayer van der Waals heterostructures of graphene/hBN/graphene. Cu and Ni atoms are deposited on the sapphire substrate and annealed to form the CuNi(111) film. Carbon atoms dissolve into the alloy film as C‐CuNi(111) on sapphire. H_2_ plasma is used to clean the alloy surface, and monolayer hBN is synthesized on top of the C‐CuNi(111) substrate. Single‐crystal monolayer graphene is then grown on top of the hBN film. The dissolved carbon atoms diffuse from C‐CuNi(111) to the interface and form the graphene layer between hBN and CuNi(111).

In this study, we synthesized wafer‐scale single‐crystal graphene/hBN/graphene multilayer van der Waals heterostructures with an AAB‐stacking mode via in situ CVD growth. First, to create an ideal substrate, we fabricated single‐crystal CuNi(111) films on a two‐inch sapphire wafer (**Figure** **1**). Carbon atoms that decomposed from methane were pre‐dissolved in the CuNi(111) film in the CVD growth chamber, and the concentration was determined according to the carbon solubility in the CuNi alloy. Then a hydrogen‐argon plasma was used to create a clean metal surface by removing the graphene islands grown on the surface of the CuNi(111) film. Single‐crystal monolayer hBN was epitaxially grown on the cleaned CuNi(111) surface for the small lattice mismatch of the two materials. Monolayer graphene was then synthesized directly on top of the hBN layer to form the graphene/hBN heterostructure. Controlling the decrease in the growth temperature facilitated the diffusion of carbon atoms pre‐dissolved in the CuNi(111) alloy, and single‐crystal monolayer graphene was grown at the interface between the hBN layer and CuNi(111) surface. Finally, high‐quality single‐crystal graphene/hBN/graphene trilayer van der Waals heterostructures were formed on the CuNi(111) substrates. Various layers of graphene under the hBN layer can be achieved by precisely adjusting the Ni components of the Cu*_x_* Ni_1‐_ *_x_* film. Raman spectroscopy and transmission electron microscopy (TEM) with selected area electron diffraction (SAED) were used to verify the crystal quality of the as‐grown graphene and hBN layers and to identify the stacking mode of the heterostructures. The proposed in situ CVD growth method can be extended to the synthesis of various multilayer graphene‐based van der Waals heterostructures.

## 2 Results and Discussion

### 2.1 Fabrication of CuNi(111) Alloy Film and Synthesis of Single‐crystal hBN

**Figure 2:**
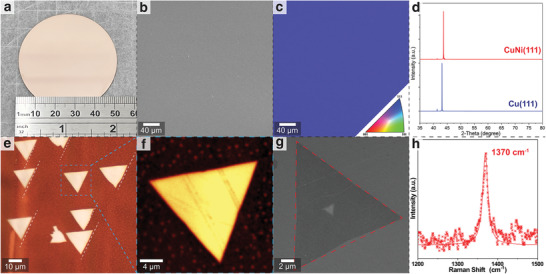
a) Photograph of the produced CuNi(111)/Al_2_O_3_(0001) wafer. b,c) SEM image and EBSD map of the surface of the CuNi(111) film. d) Typical XRD patterns of the CuNi(111) and Cu(111) films on the Al_2_O_3_(0001) wafers. e,f) Optical images of the as‐grown hBN islands on the CuNi(111) film after the oxidization treatment. g) SEM image of the triangular hBN island on the CuNi(111) film at a region different from that shown in (f). h) Typical Raman spectrum of an hBN film after being transferred onto the SiO_2_/Si substrate, with an E_2g_ mode at 1370 cm^−1^.

The Al_2_O_3_(0001) wafer was treated with an acid mixture to remove scratches and residual stress by etching the surface, producing an atomically smooth surface consisting of a terrace‐and‐step structure. Then, 450‐nm‐thick Cu followed by 50‐nm‐thick Ni were deposited on the Al_2_O_3_(0001) substrate through e‐beam evaporation to form a Cu_90_Ni_10_ alloy on Al_2_O_3_. After annealing at 1050 °C for 2 h in the CVD system with flowing hydrogen (50 sccm) and argon (350 sccm) (Figure S1 and Table S1, Supporting Information), the CuNi alloy gradually transformed to the CuNi(111) film on the two‐inch Al_2_O_3_(0001) substrate (**Figure** **2**a). The scanning electron microscopy (SEM) images showed no contrast, indicating a smooth surface with no visible grain boundaries (Figure 2b). A typical electron backscatter diffraction (EBSD) and inverse pole figure color map confirmed that the film is a single crystal (Figure 2c). The X‐ray diffraction (XRD) patterns corresponded with the EBSD results, indicating that the as‐fabricated film is a single crystal with a (111) surface. The XRD pattern of the alloy showed a slight peak shift toward a higher angle than those of an fcc Cu(111) film on sapphire (Figure 2d).

By utilizing the specific solubility of carbon in the CuNi alloy,^[^ ^33^ ^]^ methane gas was introduced into the CVD growth chamber to dissolve the carbon atoms into the CuNi alloy. Meanwhile, some graphene islands formed on the surface of the CuNi(111) film. Hence, the hydrogen‐argon plasma was used to remove the graphene islands, which created a clean surface of the CuNi(111) film. Subsequently, ammonia borane was heated to 80 °C and then introduced into the CVD growth chamber under Ar carrier gas flow, leading to the formation of single‐crystal hBN islands on the as‐fabricated CuNi(111)/sapphire substrate at 1050 °C (Figure S2, Supporting Information). The standard triangular hBN islands were aligned in a consistent orientation, indicating the high crystal quality and their ability to merge into a large‐scale single‐crystal hBN film (Figure 2e–g). Some white polymeric aminoborane nanoparticles were found around the hBN islands. These white nanoparticles were non‐crystalline BN nanoparticles of 50–100 nm diameter, which are stable under ambient conditions.^[^ ^34^, ^35^ ^]^ Figure 2h shows the typical Raman spectrum of as‐grown hBN that exhibits a clear E_2g_ mode at 1370 cm^−1^ with a full width at half maximum (FWHM) of 15.2 cm^−1^, indicating that as‐grown hBN is a high‐quality monolayer.^[^ ^23^ ^]^

### 2.2 Growth of Graphene/hBN Heterostructures on CuNi(111)

**Figure 3:**
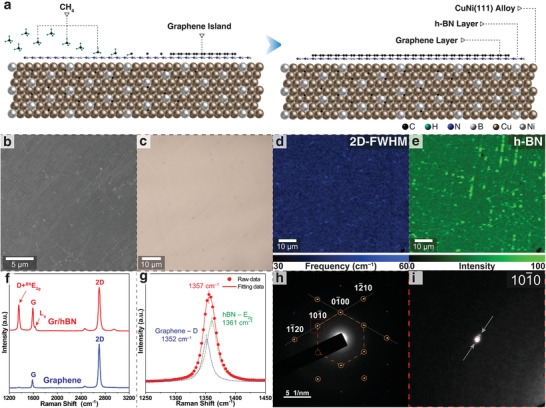
a) Atomic schematic of the top‐layered graphene growth process. b,c) SEM and optical images of the as‐grown graphene/hBN heterostructure on the CuNi(111) film. d,e) Raman maps of 2D FWHM of the graphene layer and intensity of E_2g_ of the hBN layer. f) Typical Raman spectra of the as‐grown graphene/hBN and monolayer graphene on SiO_2_/Si substrates. g) Lorentzian fit of the D and E_2g_ peaks in the spectrum of graphene/hBN in (f). h,i) SAED pattern obtained from the as‐grown graphene/hBN heterostructure. The weak spots were caused by small twist angles between graphene and hBN in a small fraction of the sample.

After growing the high‐quality hBN monolayer, we synthesized single‐crystal monolayer graphene directly onto the hBN/CuNi(111) surface to form the graphene/hBN heterostructures by re‐introducing methane into the CVD system at 1030 °C (**Figure** **3**a). This process was possible because the monolayer hBN does not inhibit the catalytic effect of CuNi(111), owing to the catalytic transparency of monolayer hBN on metal surfaces.^[^ ^28^ ^]^ To decrease the influence on the pre‐grown hBN layer, we conducted the fast growth of top‐layered graphene to let the graphene film quickly cove the hBN surface as a protective layer. The SEM image showed a uniform surface with no apparent adlayers on the as‐grown graphene/hBN heterostructure (Figure 3b). A flat area without multilayered islands was also observed in the optical image (Figure 3c). Raman mapping of the graphene 2D peak FWHM showed that the as‐fabricated graphene is a uniform monolayer (Figure 3d and Figure S3, Supporting Information).^[^ ^36^ ^]^ Raman mapping of the E_2g_ peak intensity verified that as‐grown hBN remained as a high‐quality uniform monolayer (Figure 3e). A typical Raman spectrum collected from as‐fabricated graphene/hBN exhibits two sets of Raman modes: the E_2g_ of hBN and the G and 2D peaks (at 1591 and 2699 cm^−1^, respectively) belonging to graphene, confirming the co‐existence of hBN and graphene (Figure 3f). The 2D peak observed from the heterostructure was blue‐shifted compared to the Raman spectrum from the monolayer graphene on the 300‐nm SiO_2_/Si substrate, which is consistent with the spectra of most of the previously reported graphene/hBN heterostructures.^[^ ^37^ ^]^ We suggest that this shift is caused by the change in the electronic structure induced by the interaction between the graphene and hBN layers. The Raman peak at ≈1357 cm^−1^ can be fitted and resolved by two Lorentzian peaks relating to the graphene D mode (1352 cm^−1^) and the E_2g_ (1361 cm^−1^) peak of hBN (Figure 3g), which is consistent with previous observations in the graphene/hBN heterostructures.^[^ ^38^, ^39^ ^]^ A weak and sharp peak was observed above the G‐band frequency in the spectrum, and was considered as an L_a_ peak. This peak arose from the graphene/h‐BN interaction owing to the different lattice mismatches in the two systems, indicating that the twisting angle between graphene and the hBN layer is small (<2°).^[^ ^40^, ^41^ ^]^ The FWHM of the 2D peak of as‐grown graphene was ≈39 cm^−1^, indicating that the mismatch angle between graphene and hBN is <1°.^[^ ^40^ ^]^ Thus, from the Raman spectroscopy results, the stacking mode between the hBN layer and top‐layered graphene is regarded as AA stacking. The analysis of the electron diffraction patterns of the bilayer heterostructure allows to gain a deeper understanding of the relative orientation of the two films. Therefore, we separated the as‐fabricated graphene/hBN film from the CuNi(111)/Al_2_O_3_ substrate and transferred it onto a TEM grid to collect the SAED patterns. The SAED pattern in Figure 3h shows one set of six‐fold diffraction spots, indicating the parallel alignment of graphene with h‐BN in the heterostructures (Figure 3i), which is consistent with the SAED patterns reported previously on graphene/hBN heterostructures.^[^ ^42^, ^43^, ^44^ ^]^ The SAED pattern corroborates with the Raman spectrum, indicating that as‐prepared graphene/hBN represented an AA stacking mode between layers.

### 2.3 Formation of Graphene/hBN/Graphene Trilayer van der Waals Heterostructures

**Figure 4:**
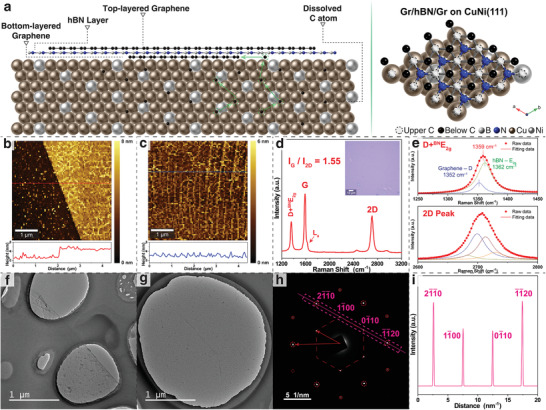
a) Atomic schematic of carbon segregation and graphene growth at the interface between the hBN layer and CuNi(111) film. b,c) AFM images of as‐grown graphene/hBN/graphene on the SiO_2_/Si substrate at b) the film edge and c) inner area. Height profiles along the marked line are plotted in the bottom inset. d) Typical Raman spectrum of the as‐grown graphene/hBN/graphene on the SiO_2_/Si substrate; the inset is the optical image. e) Lorentzian fit of the D and E_2g_ peaks (upper) and 2D peak (below) in the Raman spectrum in (d). f,g) Large‐scale TEM images of graphene/hBN/graphene on the TEM grid. h) Representative SAED pattern obtained from the as‐grown graphene/hBN/graphene film. i) Intensity profile of the diffraction spots along the magenta dashed line in the SAED pattern in (h).

By controlling the decrease of the temperature of the CVD system from 1030 to 1000 °C after growing top‐layered graphene on hBN, the previously dissolved carbon atoms in the CuNi(111) film gradually precipitated to the interface between CuNi(111) and hBN. To prevent the formation of BCN during the entire process, we optimized the hBN growth parameters to ensure that the pre‐grown hBN monolayer had fully covered the CuNi(111) surface. Considering the small lattice mismatches among graphene, CuNi(111), and hBN (3%–4% between graphene and Cu (111);^[^ ^45^ ^]^ ≈1.3% between graphene and Ni(111);^[^ ^46^ ^]^ and ≈1.6% between graphene and hBN^[^ ^47^ ^]^), under the interplay of the under‐layered single‐crystal CuNi(111) surface and upper‐layered single‐crystal hBN monolayer, the precipitated carbon atoms nucleated and epitaxially grew to form a single‐crystal monolayer graphene film at the interface, resulting in the formation of graphene/hBN/graphene trilayer van der Waals heterostructures (**Figure** **4**a). Atomic force microscopy (AFM) was used to characterize the as‐prepared van der Waals heterostructures after transferring the sample to a 300 nm SiO_2_/Si substrate (Figures S4 and S5, Supporting Information); the results indicated that the heterostructure film thickness was ≈3.9 nm (Figure 4b). A smooth surface with small surface roughness (≈0.8 nm) was observed in the inner area of the heterostructure film (Figure 4c). The typical Raman spectrum showed the G (1588 cm^−1^) and 2D (2706 cm^−1^) signals assigned to graphene and the E_2g_ signal associated with hBN (Figure 4d). The blue‐shifted 2D band compared to the Raman signal of the graphene/hBN heterostructure indicated an increase in the number of graphene layers.^[^ ^36^ ^]^

**Figure 5:**
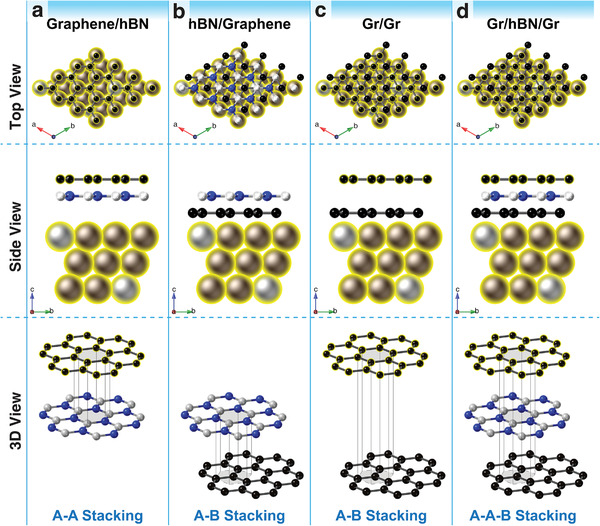
Top, side, and 3D views of atomic structures of graphene/hBN/graphene bilayer and trilayer heterostructures on the CuNi(111) substrates. The stacking modes are shown in the systems of a) top‐layered graphene and hBN, b) hBN and bottom‐layered graphene, c) top‐layered and bottom‐layered graphene, and d) top‐layered graphene, hBN, and bottom‐layered graphene. C, B, N, Cu, and Ni atoms are shown in black, white, blue, gold, and silver, respectively.

The peak at ≈1359 cm^−1^ was fitted by two Lorentzian peaks centered at ≈1352 cm^−1^ (D peak of graphene) and 1362 cm^−1^ (E_2g_ peak of hBN) (Figure 4e). The increase in the intensity of the D signal was attributed to the presence of the decomposition byproducts of borazine in the samples, such as c‐BN or BCN and BN soot during the hBN growth stage.^[^ ^48^, ^49^ ^]^ The Raman signal shows an *I*_G_/*I*_2D_ ratio of ≈1.55, and the 2D peak has an FWHM of ≈49 cm^−1^ that was fitted by four Lorentzian peaks, confirming the AB stacking mode of the top‐ and bottom‐layered graphene films in the as‐prepared heterostructures.^[^ ^36^, ^50^ ^]^ The graphene/hBN/graphene heterostructures were transferred to a TEM grid for imaging and to collect the SAED patterns (Figure 4f,g). Only one set of six‐fold diffraction patterns was observed in the SAED image (Figure 4h). The quantitative intensity analysis of the typical SAED pattern showed that the first‐order diffraction has a lower intensity than that of the second‐order diffraction, suggesting the presence of an AB stacking mode between the two graphene layers (Figure 4i).^[^ ^36^, ^51^, ^52^ ^]^ This result confirmed the presence of the AAB‐stacking mode between the layers in the as‐prepared graphene/hBN/graphene van der Waals heterostructures (**Figure** **5**). We also found that the carbon solubility in the Cu_*x*_Ni_1‐*x*_ films increases with an increase in the Ni component (1−*x*), and a higher component of carbons in the CuNi film can lead to the growth of more graphene layers at the interface, resulting in the formation of van der Waals heterostructures with multilayered graphene under the hBN film (Table S2, Supporting Information). The ability to produce different numbers of graphene layers can facilitate the fabrication of tunable‐layered graphene/hBN/graphene van der Waals heterostructures.

## 3 Conclusion

In this study, we fabricated a single‐crystalline CuNi(111) film on a sapphire wafer and subjected it to carbon dissolution to incorporate a specific concentration of carbon atoms into the CuNi(111) film. A high‐quality single‐crystal hBN monolayer was prepared on the surface of the CuNi(111) alloy after plasma cleaning. Following this, a monolayer graphene film was grown on top of hBN to form an AA‐stacked graphene/hBN heterostructure. Subsequently, a high‐quality single‐crystal graphene monolayer was formed at the interface between the hBN layer and CuNi(111) surface through a controlled decrease in the temperature of CVD growth, resulting in the formation of an AAB‐stacked graphene/hBN/graphene trilayer van der Waals heterostructure on the CuNi(111)/sapphire substrate. This study introduces a strategy for the contamination‐free in situ CVD synthesis of wafer‐scale single‐crystal graphene/hBN/graphene van der Waals heterostructures. The proposed method will provide a foundation for the development of more promising functionalized 2D materials for industrial applications.

## 4 Experimental Section

### Production of CuNi(111) Film on Sapphire Wafer

The sapphire substrate (Al_2_O_3_(0001), 2 in., c‐plane, and double‐side polished from 11‐D Tech) was cleaned using hot acetone, isopropanol, and deionized water for 5 min. A mixed acid solution (H_2_SO_4_:H_3_PO_4_ = 3:1) was used to etch the surface at 320 °C for 30 min. The sapphire was then rinsed with deionized water and cleaned using oxygen plasma. Subsequently, Cu and Ni films were deposited on the Al_2_O_3_ (0001) wafer. The resulting CuNi/Al_2_O_3_ was loaded into the CVD system for annealing at 1050 °C under H_2_ (50 sccm, 99.999%, Air Liquide) and Ar (350 sccm, 99.999%, Air Liquide) atmospheres at a pressure of 750 Torr for 2 h. After annealing, a single‐crystal CuNi (111) film was formed on the Al_2_O_3_(0001) wafer.

### Growth of hBN and Graphene

hBN was synthesized on the CuNi(111) surface in a CVD system for 40 min at 1050 °C using ammonia borane (H_3_NBH_3_, 99%, Sigma‐Aldrich) as a precursor, with H_2_ (15 sccm) and Ar (7 sccm) as the carrier gases. During the CVD growth process, the precursor was heated and maintained at 80 °C. After the growth of hBN, upper‐layered graphene was synthesized on the hBN surface in a CVD system at 1030 °C for 20 min, using a mixture of CH_4_ (10 sccm, 99.999%, Air Liquide) and H_2_ (20 sccm). After the growth of upper‐layered graphene, the chamber temperature was slowly decreased to 1000 °C over 30 min under CH_4_ (5 sccm) and H_2_ (10 sccm) gas flow. After the completion of the CVD growth process, the sample was removed from the furnace zone and quickly cooled under the same gas flow and pressure.

### Transfer of as‐Grown Heterostructures

The as‐grown heterostructures were transferred onto a SiO_2_/Si substrate using an electrochemical delamination process. The graphene/hBN/graphene heterostructure was first spin‐coated with poly‐methyl methacrylate (PMMA 950 A4, 4000 rpm for 1 min) as a protective layer; an aqueous NaOH solution was used as the electrolyte. Upon applying a constant current with CuNi(111) as the cathode, H_2_ bubbles were formed at the interface between the CuNi(111) substrate and the PMMA/graphene/hBN/graphene film, leading to the detachment of the PMMA/graphene/hBN/graphene layers from the CuNi(111)/Al_2_O_3_ substrate. After rinsing with deionized water, the stacked layers were transferred to the target substrate. Eventually, PMMA was removed using hot acetone, rinsed with isopropanol, and dried in air.

### Characterization

Raman spectra and maps were obtained by the confocal Raman spectroscopy (Alpha 300 R, WITec) using 488 and 532 nm lasers. SEM (Quattro, FEI) equipped with EBSD was used to characterize the surface morphologies of the CuNi alloy and the as‐grown 2D materials. XRD (D2 PHASER, Bruker) patterns were obtained for the Cu(111) and CuNi(111) films on the Al_2_O_3_(0001) substrates. AFM (Dimension Icon, Bruker) was used to determine the surface roughness and layer thickness. TEM images and SAED patterns were acquired using a transmission electron microscope (JEM‐2100, JEOL) at an acceleration voltage of 200 kV.

## Conflict of Interest

The authors declare no conflict of interest.

## Supporting information

Supporting Information

## Data Availability

Research data are not shared.
