# Supplementary material for: Synthesis of AAB‐Stacked Single‐Crystal Graphene/hBN/Graphene Trilayer van der Waals Heterostructures by In Situ CVD
Source: Adv Sci (Weinh). 2022 May 26;9(21):2201324. doi: 10.1002/advs.202201324 (PMC9313474; doi:10.1002/advs.202201324)
Supplement: Supplementary file 1 — Supporting Information [file ADVS-9-2201324-s001.pdf]

Synthesis of AAB-stacked Single-crystal Graphene/hBN/graphene Trilayer Van der Waals Heterostructures by *in situ* CVD

Bo Tian, Junzhu Li, Mingguang Chen, Haocong Dong and Xixiang Zhang**

[*] Corresponding-Author

B. Tian, J. Li, M. Chen, Prof. X. Zhang.
Physical Science and Engineering Division, King Abdullah University of Science and Technology (KAUST), Thuwal 23955-6900, Saudi Arabia
E-mail: bo.tian@kaust.edu.sa and xixiang.zhang@kaust.edu.sa

B. Tian, J. Li, H. Dong.
Eleven-Dimensional Nanomaterial Research Institute, Xiamen 361000, China

Keywords: van der Waals heterostructures, graphene, hBN, AAB-stacking, *in situ* CVD.

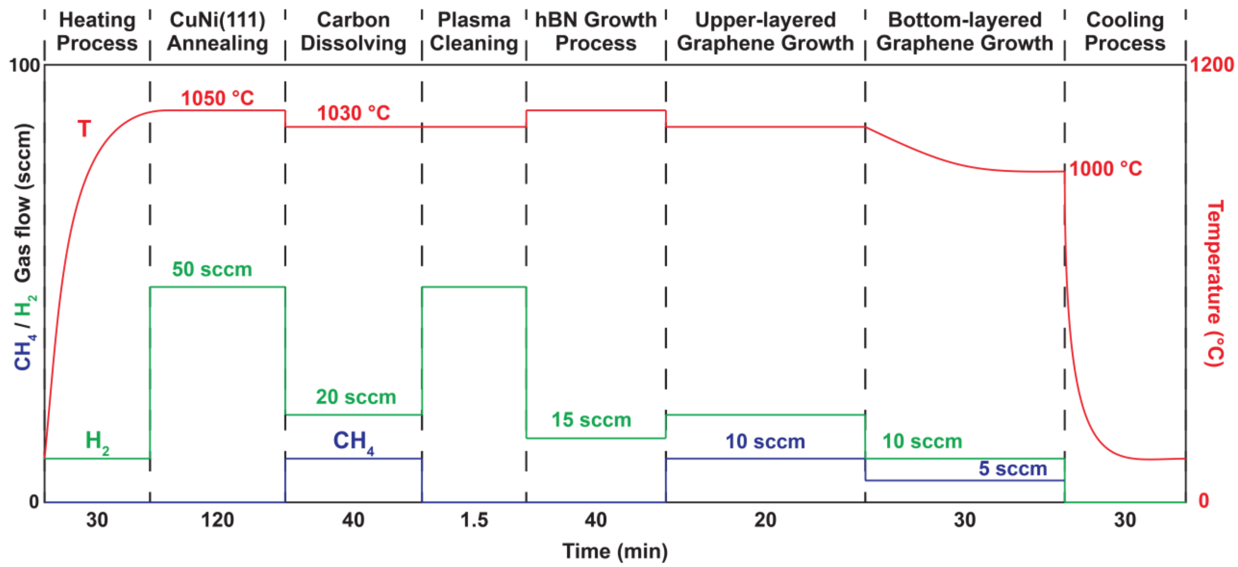

Figure S1. Diagram of CuNi annealing, hBN and graphene CVD growth, including system temperature and the gas flow of methane and hydrogen of each stage.

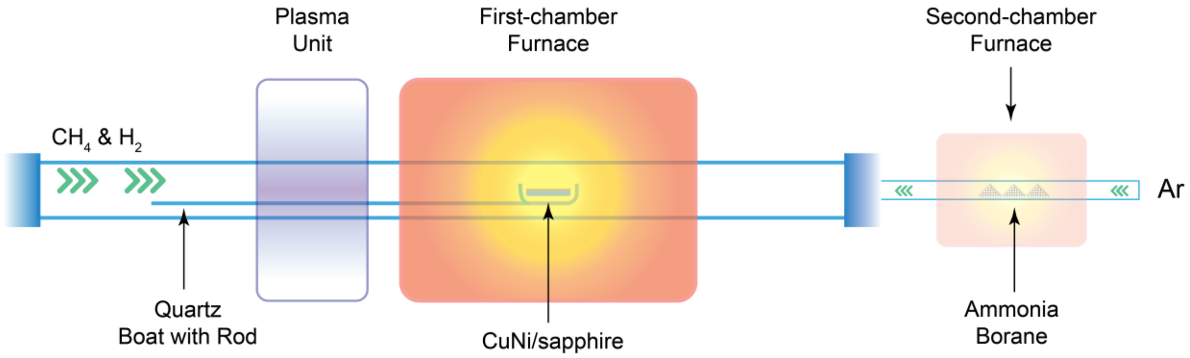

Figure S2. Schematic of the CVD system for hBN growth consists of a controlled PC, high-temperature furnace, second-chamber furnace, quartz tube, radio frequency (RF) plasma cleaning unit, load-unload quartz boat, and the CuNi(111)/sapphire substrate.

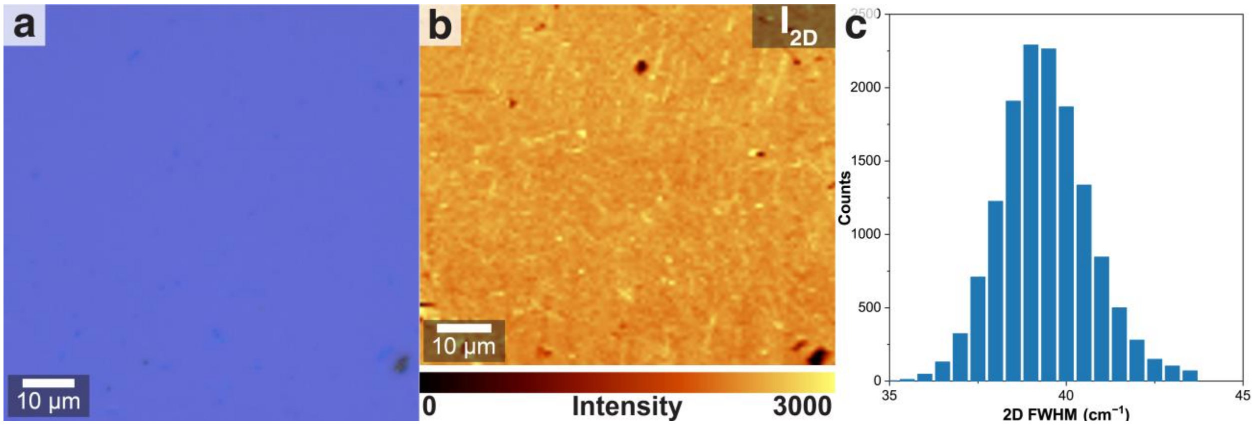

Figure S3. (a) Optical image of as-grown graphene/hBN heterostructure transferred onto SiO_2/Si substrates. (b) Raman map of the intensity of the 2D peak of as-grown graphene/hBN heterostructure. (c) 2D FWHM distribution extracted from the Raman mapping of as-grown graphene/hBN film.

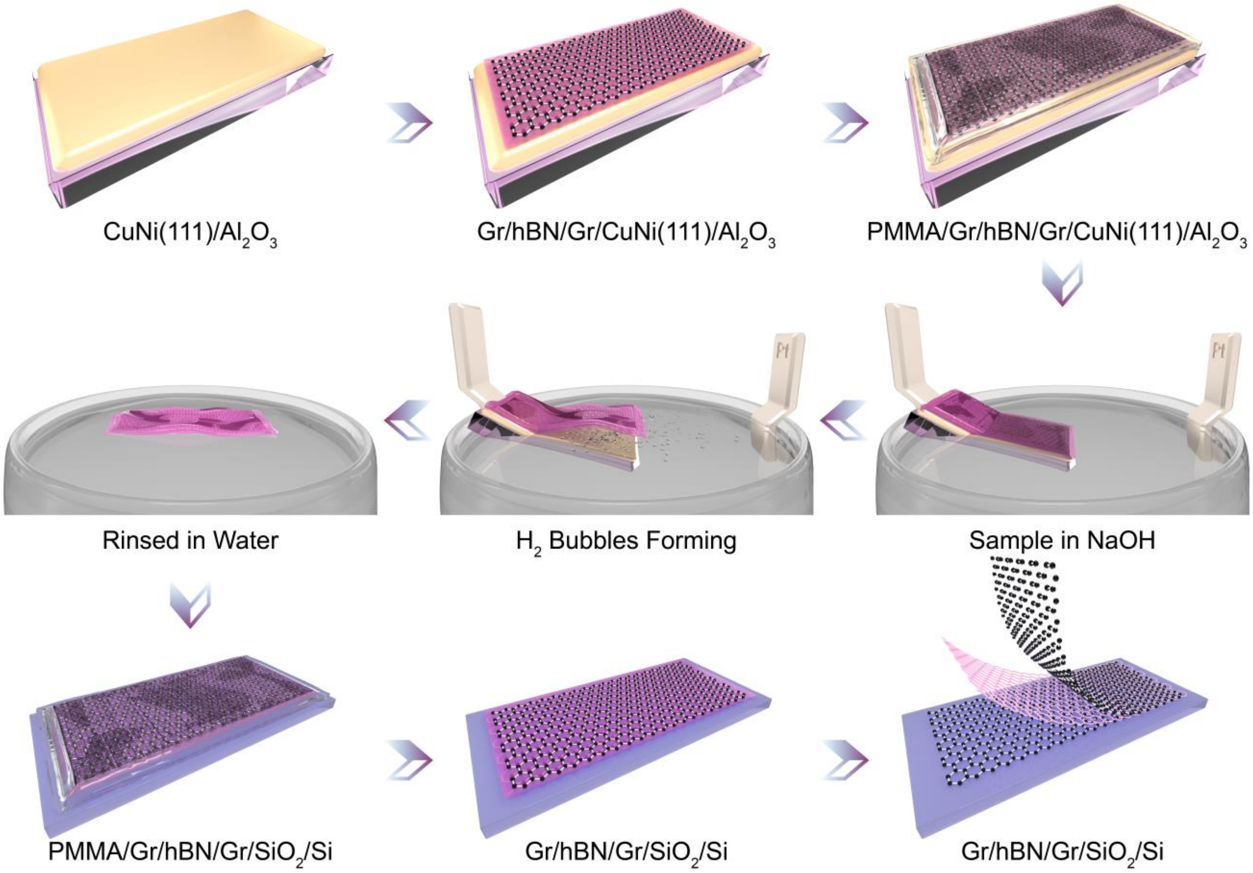

Figure S4. Schematic of the electrochemical delamination process of transferring as-grown graphene/hBN/graphene onto the SiO_2/Si substrate. The graphene/hBN/graphene heterostructure was fabricated on CuNi(111)/Al_2O_3. Then PMMA was spin-coated on the surface as the protecting layer. Then the sample was put in a NaOH aqueous solution. By applying a constant current with CuNi(111) as cathode, the H_2 bubbles formed at the interface between graphene/hBN/graphene and CuNi(111) film, leading that PMMA/graphene/hBN/graphene stack was detached from the CuNi(111)/Al_2O_3. Rinsing by deionized water, the PMMA/graphene/hBN/graphene was transferred onto SiO_2/Si substrate. Finally, the PMMA film was removed by acetone.

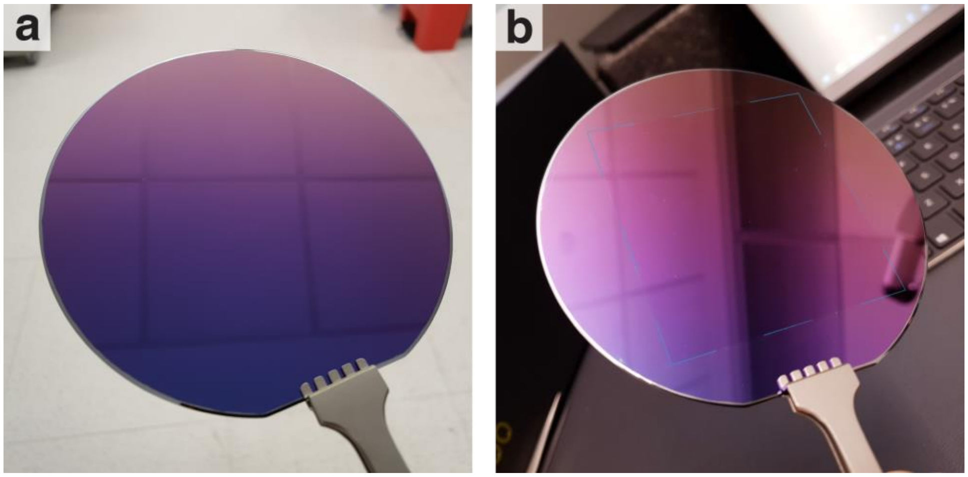

Figure S5. (a) Photograph of the bare 300 nm SiO2/Si wafer. (b) Photograph of the graphene/hBN/graphene heterostructure transferred onto the SiO2/Si substrate. The heterostructure film was cut to square by removing the scotch-tape-contacted edge area during the transfer process.

Table S1. CVD growth parameters.

Stages	CuNi(111) Annealing	Carbon Dissolving	Plasma Cleaning	hBN growth Process	Upper-layered Graphene Growth	Bottom-layered Graphene Growth
**Temp. (℃)**	1050	1030	1030	1050 | 80	1030	1000
**CH_4 (sccm)**	0	10 – 20	0	0	10	5
**H_2 (sccm)**	50	20	50	15	20	10
**Ar (sccm)**	350	0	50	7	0	0

Table S2. Graphene layers between hBN and CuNi(111) alloy in various Ni components.

**Alloy Film**	**$Cu_{95}Ni_5$**	**$Cu_{90}Ni_{10}$**	**$Cu_{85}Ni_{15}$**	**$Cu_{80}Ni_{20}$**	**$Cu_{75}Ni_{25}$**
**Graphene Layers**	< 1	~1	2 − 4	3 − 6	> 6
